# Etat des lieux des mycobactérioses atypiques au Burkina Faso: résultats d'une enquête régionale

**DOI:** 10.11604/pamj.2014.17.188.3639

**Published:** 2014-03-11

**Authors:** Sylvie Zida, Zékiba Tarnagda, Antoinette Kaboré, Dézémon Zingué, Hervé Hien, Adama Sanou, Kireopori Michel Gomgnimbou, Moumini Nouctara, Mamoudou Ouédraogo, Oumarou Ouédraogo, Sylvain Godreuil, Nicolas Méda

**Affiliations:** 1 Centre MURAZ, Bobo Dioulasso, Burkina Faso; 2 Institut de Recherche en Sciences de la Santé, Bobo Dioulasso, Burkina Faso; 3 CHU Montpellier, Montpellier, France; 4 Université de Ouagadougou, UFR/SDS, Ouagadougou, Burkina Faso

**Keywords:** Mycobactéries atypiques, complexe tuberculosis, VIH, atypical mycobacteria, tuberculosis complex, HIV

## Abstract

**Introduction:**

L’étude avait pour but de déterminer la fréquence d'isolement des mycobactéries atypiques chez les patients déclarés tuberculeux pulmonaire à microscopie positive (TPM+) au Burkina Faso.

**Méthodes:**

Il s'est agit d'une étude transversale qui s'est déroulée de mars 2011 à février 2012. Les patients TPM+ de plus de 15 ans ont été inclus dans l’étude de manière systématique dans les centres de diagnostic et de traitement (CDT) de la région sanitaire des Hauts Bassins. Une culture des mycobactéries et une identification biochimique ont été réalisées sur les échantillons des patients TPM+ La sérologie VIH a été réalisée par des tests rapides.

**Résultats:**

Une fréquence de 11% (8/73) de mycobactéries atypiques et 89,0% (65/73) de mycobactéries du complexe *tuberculosis* ont été identifiées des 73 patients TPM+ inclus dans l’étude. La tranche d’âge de 20 à 40 ans était la plus touchée par les mycobactérioses en général et constituait 48% de l’échantillon. Nous avons trouvé 12,2% (8 / 66) de patients VIH positif, 83,3% (55 / 66) de patients VIH négatif et 4,5% (3 / 66) de patients dont le statut VIH était indéterminé.

**Conclusion:**

Notre étude a mis en lumière l'implication des mycobactéries atypiques dans la pathogénie de certains patients déclarés TPM+. Le rôle des mycobactéries atypiques dans les infections pulmonaires est probablement sous estimé et devra être examiné avec plus de détails et sur un échantillon plus grand.

## Introduction

La tuberculose est une maladie infectieuse à transmission essentiellement aérienne causée par les mycobactéries du complexe *tuberculosis*. Cependant, à côté de ces mycobactéries tuberculeuses, on note la présence d'autres mycobactéries dites atypiques qui sont responsables chez l'homme de pathologies semblables à des tuberculoses appelées mycobactérioses atypiques [1]. Les infections pulmonaires à mycobactéries atypiques constituent un problème de santé publique à degré variable selon les régions du monde. En Amérique du Nord, les taux de l'infection et de la maladie variaient respectivement de 1 à 15 et de 0,1 à 2 pour 100 000 habitants. Ces études ont aussi montré une variabilité géographique de la prévalence [1].

L'incidence des mycobactérioses atypiques en France par exemple est de 0,73 cas pour 100 000 habitants chez les patients séronégatifs pour le VIH [2]. Selon une étude réalisée au Honduras en 1997, la proportion des mycobactéries atypiques parmi les cas suspects de tuberculose est estimée à 10% [3].

En Afrique, les seules études communautaires disponibles ont été réalisées en Afrique du Sud [4]. Plusieurs facteurs de risque spécifique ont été identifiés dans différentes études parmi lesquels le VIH/SIDA, les maladies chroniques à prédilection pulmonaire, le travail dans les mines, le climat chaud, un âge avancé [5]. Des études antérieures ont montré qu'une large proportion des personnes s'infecte par les mycobactéries non tuberculeuses (MNT) à partir des réservoirs environnementaux [6]. Elles posent également des difficultés diagnostiques et thérapeutiques [2].

En effet, dans les pays à ressources limitées comme le Burkina Faso, le premier outil diagnostique de la tuberculose est la microscopie dont le principe est basé sur les propriétés tinctoriales des mycobactéries. Ces propriétés tinctoriales n’étant pas spécifiques aux mycobactéries du complexe *tuberculosis*, la microscopie seule ne permet pas de conclure sur l'identité de l'espèce mycobacterienne en présence [7]. Les manifestations cliniques et radiologiques de la maladie pulmonaire à mycobactéries atypiques sont subtiles et s'apparentent souvent à la tuberculose [8]. Les conséquences sont des échecs de traitements et des rechutes augmentant la mortalité chez les malades déclarés tuberculeux.

Des lacunes de connaissances existent sur la fréquence des mycobactéries atypiques en Afrique subsaharienne dont le Burkina Faso. Le programme national tuberculose du Burkina Faso repose sur le diagnostic et le traitement des cas TPM+ et TPM-. Les cas de mycobactérioses atypiques échappent au traitement car aucune recommandation de diagnostic et de traitement n'est préconisée pour leur prise en charge[9]. Une determination de la fréquence des ces mycobatéries atypiques parmi les cas déclarés TPM+ pourrait succiter la reflexion sur les stratégies de diagnostics et de traitements de la tuberculose et des mycobactérioses atypiques. L'objectif de cette étude est de déterminer la fréquence des mycobactéries atypiques parmi les patients déclarés TPM+, afin d'en améliorer le diagnostic et le traitement.

## Méthodes

**Population d’étude et critères d'iligibilité:** nous avons conduit une étude transversale de mars 2011 à février 2012 dans la région sanitaire des Hauts Bassins au Burkina-Faso. Le recrutement des patients a été réalisé dans 8 CDT de la région sanitaire des Hauts Bassins. Ces sites étaient ruraux et urbains. La population d’étude était constituée de tous les patients dépistés positifs à la tuberculose dans les 8 sites CDT. Était inclus dans l’étude tout patient déclaré TPM+ ayant plus de 15 ans, et ayant donné son consentement à participer à l’étude.

**Echantillonnage et taille de l’échantillon:** nous avons procédé à un échantillonnage systématique des patients dans les différents sites CDT pendant toute la période d’étude ( 12 mois) Selon les prévisions des cas de tuberculoses dans les sites pendant la période, nous nous attendons à inclure 100 patients déclarés TPM+.

**Techniques et méthodes de collecte des données:** des entretiens individuels ont été réalisés avec les patients. Un questionnaire standardisé a été utilisé pour collecter les informations sur les caractéristiques socio démographiques des patients et le statut VIH.

**Méthode de traitement des échantillons:** les échantillons de patients TPM+ étaient transférées des sites CDT au laboratoire de mycobactériologie du Centre MURAZ. Des échantillons d'expectorations étaient collectés. Un prélèvement de 5 mL de sang sur tube EDTA a été réalisé chez tous les patients pour le test du VIH après un counceling. Les échantillons d'expectorations positifs arrivés au Centre MURAZ étaient décontaminés par la méthode de Pétroff à la soude [6]. Après la décontamination les échantillons ont été ensemencés sur milieu solide de Loewenstein-Jensen (LJ) et LJ enrichi en pyruvate. Les cultures positives ont fait l'objet d'une identification biochimique. Le test au nitrate et la recherche de la production de la catalase à 22°C et 68°C ont été utilisés pour identifier et différencier les mycobactéries du complexe tuberculosis et les mycobactéries atypiques. La sérologie VIH des patients a été effectuée à l'aide de tests rapides Détermine^®^ HIV et Génie III^®^ HIV1 /HIV2 avec le plasma des patients.

**Collecte et analyse des données:** les caractéristiques socio démographiques et cliniques des patients ont été décrites ( âge moyen, sexe ratio); La proportion des mycobactéries atypiques a été calculée, Une analyse univariée a été réalisée en comparant la proportion des mycobactéries atypiques selon l’âge, le sexe, le statut VIH. Le test de Chi 2 a été utilisé pour les comparaisons. Quand les conditions n’étaient pas satisfaites le test de Ficher Exacte a été utilisé. Le seuil de significativité de 5% a été utilisé pour tous les tests statistiques.

**Considérations éthiques et réglementaires:**cette étude a reçu un avis favorable à sa mise en ‘uvre par le comité d′éthique institutionnel du Centre Muraz le 15 juin 2010 (N°A012-2010/CE-CM) et par le Comité d′éthique pour la recherche en santé le 07 juillet 2010 (Délibération N°2010-49) Cette étude a reçu l'autorisation du comité d’éthique de recherche en santé du Burkina Faso Cette étude a été réalisé au décours d'une recherche de l’épidémiologie de la transmission de la tuberculose au Burkina Faso financé par l'agence nationale de recherches sur le SIDA et les hépatites virales (ANRS). L'approche des patients a été faite suivant les directives du programme national de lutte contre la tuberculose (PNT) du Burkina Faso [9].

## Résultats

### Caractéristiques de la population d’étude

La population d’étude était constituée de 73 patients déclarés tuberculeux pulmonaire à microscopie positive (TPM+). Ces 73 TPM+ avaient une culture positive et une identification biochimique interprétable. Le sex-ratio des patients était de 1,4 avec 71,2% d'hommes et 28,8% de femmes. L’âge moyen des patients était de 37,8 ± 15,4 ans.

### Fréquence et distribution des mycobactéries atypiques

Chez les 73 patients TPM+, nous avons isolé 89% (65/73) de mycobactéries du complexe *tuberculosis* et 11% (8/73) de mycobactéries atypiques. Les hommes représentaient 72,3% (47/65) des patients chez qui, au moins, une mycobactérie du complexe *tuberculosis*, a été isolée et 62,5% (5/8) des patients pour les mycobactéries atypiques. Le Tableau 1 représente la fréquence des mycobactéries tuberculeuses et atypiques en fonction du sexe. Le Tableau 2 représente les fréquences des mycobactéries du complexe *tuberculosis* et des mycobactéries atypiques en fonction de l’âge des patients. Les patients qui avaient un âge compris entre 20 et 40 ans étaient les plus infectés les mycobactéries tuberculeuses et atypiques (48%). Sur les 73 patients, nous avons pu réaliser la sérologie VIH de 66 patients. Nous avons trouvé 12,2% (8 / 66) de patients VIH positif, 83,3% (55/66) de patients VIH négatif et 4,5% (3 / 66) de patients dont les résultats sont indéterminés. La prévalence du VIH était de 12, 1% (7/58) dans le groupe des patients avec mycobactéries tuberculeuses et de 12,5% (1/8) dans le groupe des patients avec mycobactéries atypiques (p=0,73). Dans le groupe des patients infectés par le VIH, nous avons trouvé 12,5% (1/8) de mycobactéries atypiques et dans le groupe des patients séronégatifs, nous retrouvons 12,7% (7/55) d'isolement de mycobactéries atypiques.

**Tableau 1:** Fréquence des mycobactéries tuberculeuses et atypiques en fonction du sexe des patients

Sexe	Groupe de mycobactéries			
	Complexe *tuberculosis*		Mycobactéries atypiques	
	N	%	N	%
Masculin	47	72,3	5	62,5
Féminin	18	27,7	3	37,5
Total	65	100,0	8	100,0

**Tableau 2:** Fréquence des mycobactéries du complexe *tuberculosis* et des mycobactéries atypiques en fonction de l’âge des patients

Age	Groupe de mycobactéries					
	Complexe *tuberculosis*		Mycobactéries atypiques		Total	
	N	%	N	%	N	%
<20	7	10,8	3	37,5	10	13,6
20-40	33	50,8	2	25,0	35	48,0
>40	25	38,4	3	37,5	28	38,4
Total	65	100,0	8	100,0	73	100,0

## Discussion

Nous avons notés une fréquence de 11% de mycobactéries atypiques parmi les cas déclarés TPM+. La fréquence de TPM+ à complexe *Myctobacterium tuberculosis* était de 89% (65/73). Une étude sur les infections mycobactériennes menée au Burkina Faso en 2001 par Ouédraogo et coll. a rapporté une proportion de 6% de mycobactérioses atypiques (5 / 82) [10]. La proportion plus élevée de notre étude peut s'expliquer par l'augmentation constante des mycobactérioses à travers le monde rapporté par Casabona M. et coll. en 2004 et Glassroth J. et coll. dans une étude multicentrique en 2008 [6, 11]. Cette augmentation s'explique par l'amélioration des conditions de diagnostic et l'intérêt porté à ces mycobactérioses atypiques. Une autre étude menée par Miotto P. et coll. en 2009 toujours au Burkina Faso sur les cas chroniques de tuberculose a révélée une fréquence de 12% [12]. Des fréquences similaires de 11% et 10% ont été aussi retrouvée respectivement en Zambie en 2009 par Buijtels P. et coll. [13] et au Honduras en 1997 par Lelany Pineda-Garcia L. et coll [3].

Notre étude a montré que La tranche d’âge de 20 à 40 ans est la plus touchée par les mycobactéries tuberculeuses et atypiques avec 48%. Cela peut s'expliquer par le fait que la population du Burkina Faso est à majorité jeune. Cette tendance a été notée par une étude similaire réalisée au Burkina Faso en 2001 par Ouédraogo M. et coll [10]. Nous avons noté également que les hommes sont les plus touchés et représentent 72,3% du groupe de patients infectés par les mycobactéries du complexe *tuberculosis* et 62,5% du groupe infecté par les mycobactéries atypiques. Cette tendance a été également observée dans une étude menée en Grèce en 2008 par Gerogianni I. et coll. [14] qui rapporte 68,7% d'hommes dans le groupe complexe *tuberculosis* et 67% d'hommes dans le groupe mycobactéries atypiques. Cette étude a montré que 12,2% (8 / 66) de patients étaient VIH positif. Ce chiffre s'explique par le fait que la tuberculose est la cause majeure de morbidité et de mortalité dans les populations où la prévalence du VIH est élevée [9]. Ce taux se rapproche de celui du PNT du Burkina Faso qui rapporte une séroprévalence de 15% parmi les patients TPM+ [9]. Une autre étude réalisée au Honduras par Pineda-Garcia L. et coll. révèle un taux de séroprévalence de 14% (11/77) [3]. La fréquence d'isolement de mycobactéries atypiques chez les patients séropositifs pour le VIH (12,5%) ne diffère pas significativement de celui des malades séronégatif pour le VIH (12,7%) avec p=0,73. Ces résultats sont similaires à ceux trouvés par Ouédraogo M. et coll. [10] dans leur étude sur les infections mycobactériennes et sérologie VIH au Burkina Faso en 2001.

Cependant notre étude a quelques limites au niveau des effectifs faibles qui pourraient ne pas permettre de mettre en évidence des différences significatives dans nos comparaisons. La restriction de nos critères d'inclusion aux cas de TMP+ uniquement est également une limite et l'inclusion des TPM- pourrait élever la fréquence des mycobactréries atypiques. L'isolement des mycobactéries atypiques dans cette étude met en évidence le rôle pathogène et l'implication des mycobactéries atypiques chez les patients déclarés TPM+. La fréquence de 11% que nous avons notée est considérable quand on prend en compte les erreurs diagnostiques et thérapeutiques dont les conséquences sont lourdes pour les patients. Le rôle des mycobactéries atypiques dans les infections pulmonaires est probablement sous estimé et doit être examiné avec plus de détails et sur un échantillon plus grand. Lors de l'isolement d'une mycobactérie atypique d'un prélèvement, une interprétation critique du résultat est nécessaire, elle confrontera les données cliniques et radiologiques aux données microbiologiques. De ce fait l'incrimination d'une mycobactérie isolée dans les expectorations dans une pathologie doit suivre certains critères établis par l'American Thoracic Society [15]. Notre étude ne remplie pas tous ces critères.

Au Burkina Faso comme dans la plupart des pays en développement, le diagnostic de la tuberculose est basé sur la microscopie. Cela signifie que les 11% des patients TPM+ chez qui on a isolé des mycobactéries atypiques ont été soumis à un traitement antituberculeux alors que les mycobactéries atypiques ne sont pas sensibles à la plus part des antituberculeux classiques [6]. Ces patients traités à tort par les antituberculeux seront probablement en échec de traitement.

## Conclusion

Cette étude démontre l'importance de la culture et de l'identification des mycobactéries en complément de la microscopie dans le diagnostic de la tuberculose. Elle nous interpelle donc quant à l'amélioration du diagnostic différentiel et de la prise en charge de la tuberculose et des mycobactérioses atypiques.
